# Epidemiological Features of* Clostridium difficile* Colonizing the Intestine of Jordanian Infants

**DOI:** 10.1155/2017/2692360

**Published:** 2017-12-20

**Authors:** Eman N. Abu-Khader, Eman F. Badran, Asem A. Shehabi

**Affiliations:** ^1^Department of Pathology-Microbiology and Forensic Medicine, The University of Jordan, School of Medicine, Amman, Jordan; ^2^Department of Pediatrics, Jordan University Hospital, Amman, Jordan

## Abstract

*Clostridium difficile* is commonly found in the intestine of infants without causing any disease. This study investigated the most important epidemiological features of* C. difficile* strains colonizing intestine of Jordanian infants. A total of 287 fecal samples were collected from infants admitted to the Jordan University Hospital (JUH) over the period of 2015. Samples were cultured for* C. difficile* and their growth was identified using microbiological culture and PCR. The overall* C. difficile* colonization rate among hospitalized and nonhospitalized infants was 37/287 (12.9%). Neonates were less colonized than other infants (8.7% verses 19.5%). Colonization of the infants with* C. difficile* toxigenic strains (TcdA and TcdB) was observed in 54% of the isolates, whereas those colonized with nontoxigenic strains were 46% and only one isolate was positive for binary toxin. Breast feeding of infants is a significant factor associated with decreased colonization with* C. difficile*. All* C. difficile* strains were susceptible to vancomycin and metronidazole, while high resistance rate to ciprofloxacin (78.4%) and less resistance rate to erythromycin (29.7%) were detected among the isolates. The results showed that 40.5% of the isolates carried mutated* gyrA* and* gyrB *genes which have cross-resistance to ciprofloxacin and moxifloxacin. This study represents useful epidemiological features about* C. difficile* colonizing intestine of infants living in a developing country.

## 1. Introduction

*Clostridium difficile *is a major cause of nosocomial antibiotic-associated diarrhea due to the production of toxins A and B.* C. difficile *infection (CDI) can result in asymptomatic carriage, mild diarrhea, or pseudomembranous colitis (PMC). CDI can be associated with significant morbidity, mortality, and healthcare costs in hospitalized patients [1, 2]. Increased incidence of CDI is coupled with more serious clinical presentation; especially mortality among patients ranges from 24 to 50% [3–5].

The rate of* C. difficile* colonization among newborns varies widely (2.5–90%) [1], mainly during the first 2 years of life and usually in an asymptomatic manner [6, 7]. A recent study from USA reported increased rate of pediatric CDI-related hospitalization over the period 1997–2006 [8]. Several hypotheses have been reported to explain asymptomatic state of* C. difficile* in newborns. These include the competitive intestinal colonization by nontoxigenic strains, the immaturity of the immune system, possible absence of toxin receptors in the intestinal tract, modulation of toxin production by the infant microbiota, and toxin neutralization by maternal antibodies [6, 9].

Transmission of* C. difficile* occurs primarily in healthcare facilities, where exposure to antimicrobial drugs and environmental contamination by its spores are causing mostly nosocomial infection [10, 11].

This study investigated the occurrence rate of* C. difficile* intestinal colonization and most important epidemiological factors associated with its presence in intestine of Jordanian infants.

## 2. Patients and Methods

### 2.1. Study Population

This prospective convenience sampling study was conducted at the Pediatric Department/Jordan University Hospital (JUH) over a period of 8 months from March 2015 through October 2015. The study was approved by the School of Medicine and the School of Graduate studies at The University of Jordan. A total of 287 stool fecal samples were obtained from 151-male and 136-female infants aged one year or less.

Biographical and clinical data which were obtained from each infant included age, gender, name, duration of hospitalization, presence of diarrhea, hospital length of stay, mode of delivery, type of feeding, and antibiotics treatment at the time of sampling (Table 1).

### 2.2. Ethical Permission

A permission was also obtained from the Ethical Review Board (ERB) at the Jordan University Hospital (JUH), permission number 75/2015. Verbal consent was obtained from all mothers of infants after explaining the aim of the study.

### 2.3. Culture and Identification of* C. difficile*

One fecal sample from each patient was collected during investigation in outpatients' clinic or directly after admission to hospital, using sterile prewetted cotton swabs in 0.85% normal saline. All fecal specimens were sent within 2-3 hrs to research microbiology labs. The specimens were first treated by absolute ethanol (v/v) for 1 h before inoculation into* Clostridium difficile *moxalactam-norfloxacin agar plates (CDMN, Oxoid, England) which was supplemented with 7% (v/v) defibrinated horse blood. Culture plates were incubated for 48 hours at 37°C under anaerobic condition, and a control reference strain of* C. difficile* (NCTC 11204) was included. All suspected colonies resemble* C. difficile* in their appearance which were used for Gram and spore stains and later confirmed by Remel RapID ANA II system (Remel Inc., Lenexa, KS, USA). All* C. difficile* isolates were frozen at −70°C in brain-heart infusion broth (BHIB) with 20% glycerol for further antimicrobial susceptibility test and characterization of their potential toxins genes.

### 2.4. Antimicrobial Susceptibility Testing

All* C. difficile* isolates were tested using *E*-tests (Oxoid, England) for vancomycin, metronidazole, ciprofloxacin, and erythromycin according to guidelines of CLSI (2015) [12]. A quality control* C. difficile* strain (NCTC 11204) was included.

### 2.5. Molecular Methods

*C. difficile* DNA was prepared using a single colony by boiling at 95°C for 10 min in water bath. The identity of* C. difficile* isolates was confirmed by amplification of the 16S rRNA gene using* C. difficile* specific primers (PG48 and B) [13]. PCR reactions were used for the detection of* C. difficile* toxin genes A and B (*TcdA* and* TcdB*) and to detect genes encoding the enzymatic* (cdtA)* and binding* (cdtB)* components of the binary toxin as described by Terhes et al. [14]. Mutation detection in* gyrA and gyrB* genes was carried out using PCR as reported by Dridi et al. [15].

### 2.6. Statistics

All data analyses were carried out using SPSS version 20. *χ*^2^-test was used for statistical analysis. *P* ≤ 0.05 was considered statistically significant.

## 3. Results

### 3.1. Characteristics of Infants

Table 1 shows important characters from all examined infants with positive or negative* C. difficile *fecal isolates according to their age group, admission to intensive care unit (INCU) or outpatients department (OPD), sex, diarrhea, duration of hospitalization, type of feeding, birth weight (BWT), mode of delivery, and current antibiotic treatment.

### 3.2. Detection of* C. difficile*

A total of 37/287 (12.9%) of* C. difficile* isolates were recovered from infants aged ≤1 year, of these 20/37 (54.1%) were toxigenic strains. All isolates were confirmed using 16S rRNA gene-targeted PCR.

### 3.3. Detection of* C. difficile* Toxigenic Genes

Table 2 shows that 13/37 (35.1%) carried both genes of toxin A* (TcdA)* and B* (TcdB)*, whereas 17/37 (45.9%) of isolates were negative for toxin A and B genes. Only one isolate (2.7%) was positive for binary toxin genes (*TcdA* and* TcdB*) (Table 2). Successful sequencing (99%) of the* cdtA* and* cdtb* genes was used to confirm the identity of one isolate that was positive for the binary toxin genes (Macrogen, Korea). The sequence reads were aligned manually using the online software NCBI BLAST.

### 3.4. Antimicrobial Susceptibility

All* C. difficile* isolates were 100% susceptible for both vancomycin and metronidazole as shown by their MICs breakpoint, whereas the MICs for ciprofloxacin and erythromycin showed that 29/37 (78.4%) and 11/37 (29.7%) of the isolates were resistant, respectively. Fluoroquinolone resistance-determining mutated gene (*gyrA *and* gyrB*) were present in 15/37 (40.5%) among the isolates (Table 3).

## 4. Discussion

This study demonstrates relatively low colonization rate of* C. difficile* (12.9%) in stools of hospitalized and nonhospitalized infants aged ≤1 year. Toxigenic* C. difficile* accounted for 54.1% of the isolates (Tables 1 and 2). A prospective study performed in France over 18-month period has found that the* C. difficile *colonization of French infants aged between 0 to 2 years was 33.7%, and the colonization rate by a toxigenic strain was 7.1% which is slightly less than that in our study [9]. A recent study from USA reported that the rate of pediatric CDI-related hospitalizations increased from 7.2 to 12.8% from 1997 to 2006; the lowest rate was observed in newborns (0.5%), while incidence for children aged <1 year and those aged 5–9 years were 32% [8]. A study in Japan reported that the carriage rate of toxigenic* C. difficile* in neonates was very low (2.5%) but was increased to 84.4% in infants under 2 years of age [16].

The reason why the incidence rates of* C*.* difficile* among infants differ widely between countries remains questionable. However, the rate of colonization in infants may be due to the low capacity of the infant gut flora to suppress growth of* C. difficile* or due to the absence of toxin receptors in the infantile gut mucosa [17]. Additionally, infants are more frequently colonized than adults, but they rarely develop* C. difficile *disease during the first year of their life [18–20].

The present study shows that infants receiving formula mixed milk were significantly more associated (*P* < 0.012) with* C. difficile* colonization than breast milk (Table 1). This finding was similar to other recent studies [6, 7, 19]. Otherwise, this study showed that there was no significant correlation (*P* > 0.05) between* C. difficile* colonization and certain neonatal conditions related to gender, gestational age, birth weight, mode of delivery, hospital length of stay, presence of soft stools, and antibiotic treatment.

In Jordan, most previous studies on* C. difficile* infection and colonization have involved mostly adult patients. A study performed at the Jordan University Hospital in 2007 found 13.7% prevalence rate of toxigenic* C. difficile* isolates among adult hospitalized patients as proved by the presence of positive culture/toxin genes or both and in association with diarrhea, but there was no single case found among children [21]. The current study indicates that there was no correlation between positive colonization of toxigenic* C. difficile* in infants and presence of soft stools, previous antibiotic treatment, or inflammatory bowel disease, and overall our findings were similar to that of other investigators [18, 19]. A recent multicenter study done in three different private Jordanian hospitals in Amman over a period of 8 months showed high prevalence rate (92.4%) of positive* C. difficile *toxins among adults as well as older patients with a prolonged hospital stay and comorbidities [22]. Additionally, a recent study indicated that infants are widely colonized by nontoxigenic* C. difficile *strains and their early intestinal colonization with toxigenic strains originated from adults [9].

This study shows that all 37* C. difficile* isolates were susceptible to vancomycin and metronidazole and were highly resistant to ciprofloxacin (78.4%) and moderate resistant to erythromycin (29.7%) (Table 3). These results are similar to some extent to a previous study published from our hospital [21] which showed that all* C. difficile* isolates were highly susceptible to vancomycin and metronidazole, while moderate resistant rate was found to ciprofloxacin. These findings are also much related to those of other European studies [15, 23, 24]. It is also important to mention here that fecal* E. coli* isolates from Jordan infant and adults were highly resistant to ciprofloxacin, and it is well known that Jordan physicians are extensively using fluoroquinolones in treatment of urinary and respiratory tract infections [25–27].

In conclusion, this study presents important epidemiological data about occurrence of toxigenix* C. difficile* in intestines of hospitalized and nonhospitalized infants living in a Middle East country.

## Figures and Tables

**Table 1:** Demographic characteristics of 287 infants with positive and negative *C. difficile* culture.

Variables	Number (%) of positive * C. difficile*	Number (%) of negative * C. difficile*	*P* value
*Age by group*			
1 day–≤30 days	15 (8.7)	157 (91.3)	0.050
>1 month–≤1 year	22 (19.5)	93 (80.5)	
*Gender*			
Male	18 (11.9)	133 (88.1)	0.605
Female	19 (14.0)	117 (86.0)	
*Hospital ward*			
NICU^*∗*^	0 (0.0)	50 (100)	0.001
OPD	37 (15.6)	200 (84.4)	
*Antibiotics treatment*			
Yes	10 (10.5)	85 (89.5)	0.400
No	27 (14.1)	165 (85.9)	
Presence of diarrhea^*∗∗*^			
Yes	3 (23.1)	10 (76.9)	0.262
No	34 (12.4)	240 (87.6)	
*Hospital length of stay*			
1–7 days	14 (14.1)	85 (85.9)	0.062
8–30 days	1 (2.0)	49 (98.0)	
>30 days	2 (15.4)	11 (84.6)	
*Gestational age*			
<32	0 (0.0)	12 (100)	0.420
32–36	9 (14.3)	54 (85.7)	
37–39	16 (11.6)	122 (88.4)	
>39	12 (16.2)	62 (83.8)	
*Birth weight*			
≤2500	16 (16.2)	83 (83.8)	0.230
>2500	21 (11.2)	167 (88.8)	
*Mode of delivery*			
Normal vaginal delivery	20 (13.8)	125 (86.2)	0.654
Cesarean section	17 (12.0)	125 (88.0)	
*Type of feeding*			
Breast	4 (5.1)	74 (94.9)	0.012
Formula and mix	33 (15.8)	176 (84.2)	

^*∗*^Included 50 newborns which were admitted to neonatal intensive care unit (NICU).^*∗∗*^Mostly soft stools without any clinically significant gastrointestinal symptoms or diarrhea during the collection of the specimens.

**Table 2:** Toxigenic profile of 37 *C. difficile* isolates.

Toxin profile	Number of isolates (%)
A+ B+	13 (35.2)^*∗*^
A+ B−	1 (2.7)
A− B+	6 (16.2)
Negative A & B	17 (45.9)^*∗∗*^

Total (%)	37 (100)^*∗∗∗*^

^*∗*^Only one isolate was positive for binary toxin genes as confirmed by sequencing; ^*∗∗*^54.1% of the *C. difficile *isolates were toxigenic strains; ^*∗∗∗*^37/287 (12.9%) were intestine colonized with *C. difficile*.

**Table 3:** Antimicrobial MIC results of 37 *C. difficile* isolates.

Antimicrobial agents	MIC50 (*µ*g/ml)	MIC90 (*µ*g/ml)	Resistance breakpoint (*µ*g/ml)	MIC (*µ*g/ml) range	Number (%) of resistance
Ciprofloxacin^*∗*^	5.3	9.6	8	3.0–16.0	29 (78.4)
Erythromycin	0.42	0.75	8	0.25–2.0	11 (29.7)
Vancomycin	0.89	1.60	32	1.0–4.0	Null
Metronidazole	0.09	0.16	32	0.023–0.25	Null

^*∗*^A total of 40.5% of isolates were positive for both mutated *GyrA *and* GyrB genes*.
